# Supplementary material for: Construction of Microporous Zincophilic Interface for Stable Zn Anode
Source: Molecules. 2023 Jun 15;28(12):4789. doi: 10.3390/molecules28124789 (PMC10302738; doi:10.3390/molecules28124789)
Supplement: Supplementary file 1 [file molecules-28-04789-s001.zip › molecules-2397827-supplementary.pdf]

Supplementary Information

Construction of microporous zincophilic interface for stable Zn anode

Xin Yang [1], Tie Shu [1], Haoyu Huang [2], Hongquan Yi [2], Yanchi Zhang [2], Wei Xiao [2], Liang Li [3], Yuxin Zhang [4], Minghao Ma [5], Xingyuan Liu [6] and Kexin Yao [7,*]

1 School of Chemistry and Chemical Engineering, Chongqing University, Chongqing 400044, China; xyang0610@163.com (X.Y.); shutie950112@163.com (T.S.)
2 Undergraduate School, Chongqing University, Chongqing 400044, China; 17815041238@163.com (H.H.); 19132050489@163.com (H.Y.); 15772046781@163.com (Y.Z.); 13368183178@163.com (W.X.)
3 Department of Sciences and Engineering, Sorbonne University Abu Dhabi, Abu Dhabi P.O. Box 38044, United Arab Emirates; liang.li@sorbonne.ae
4 College of Material Science and Engineering, Chongqing University, Chongqing 400044, China; zhangyuxin@cqu.edu.cn
5 Hang Tian School Affiliated to HSXJTU, Xi'an 710043, China; minghaomacqu@163.com
6 Chongqing Joint School of Famous Schools, Chongqing 400030, China; wenqin@cqu.edu.cn
7 State Key Laboratory of Coal Mine Disaster Dynamics and Control, Multi-Scale Porous Materials Center, Institute of Advanced Interdisciplinary Studies, School of Chemistry and Chemical Engineering, Chongqing University, Chongqing 400044, China
* Correspondence: kexinyao@cqu.edu.cn

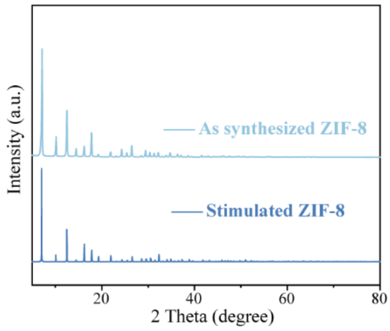

Figure S1. The XRD pattern of ZIF-8

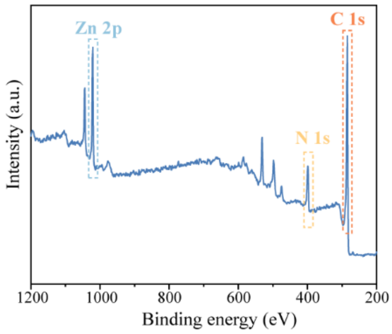

Figure S2. The wide-scan XPS spectrum of ZOCC

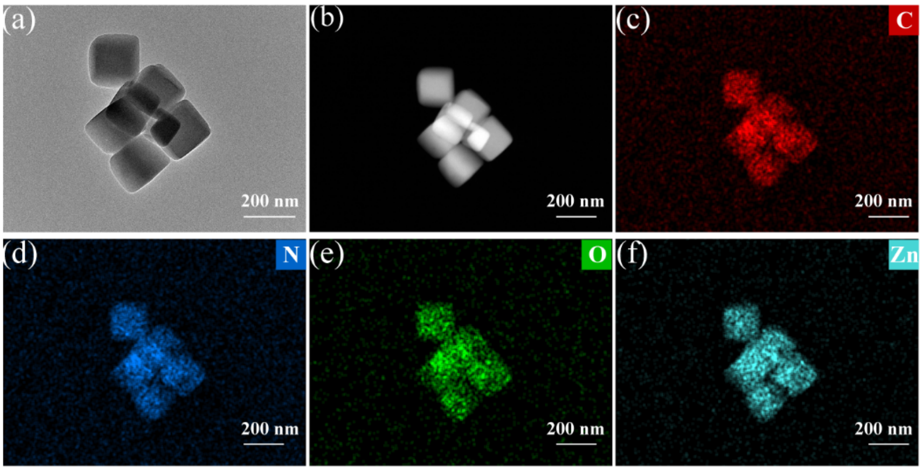

Figure S3. (a) TEM image of ZIF-8; (b) HAADF-STEM image of ZIF-8; Corresponding elemental mapping of (c) C; (d) N; (e) O; (f) Zn

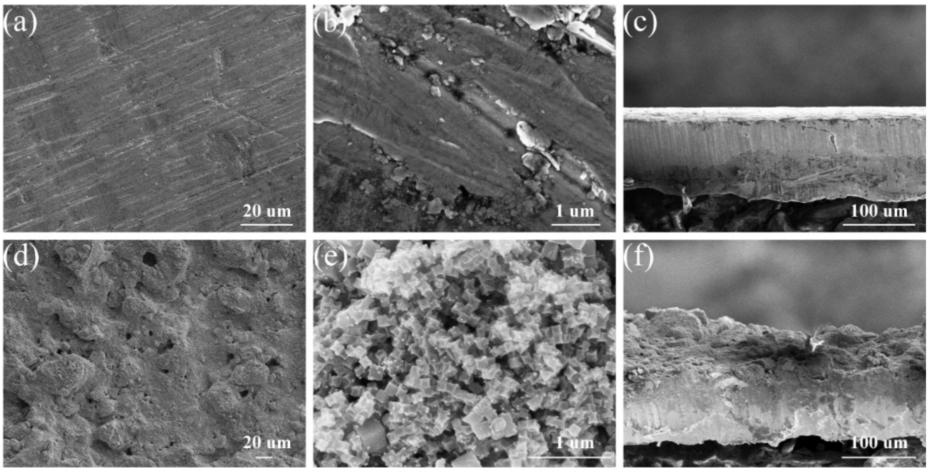

Figure S4. The surface morphology of bare Zn (a-b) and ZOCC@Zn (d-e) at different magnifications; The cross-sectional SEM images of bare Zn (c) and ZOCC@Zn (f)

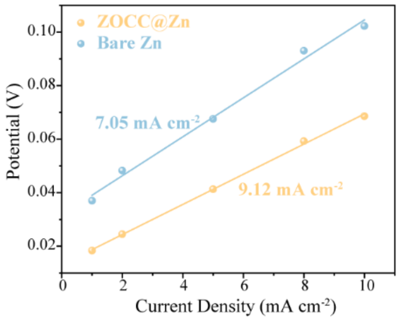

Figure S5. Exchange current density plot

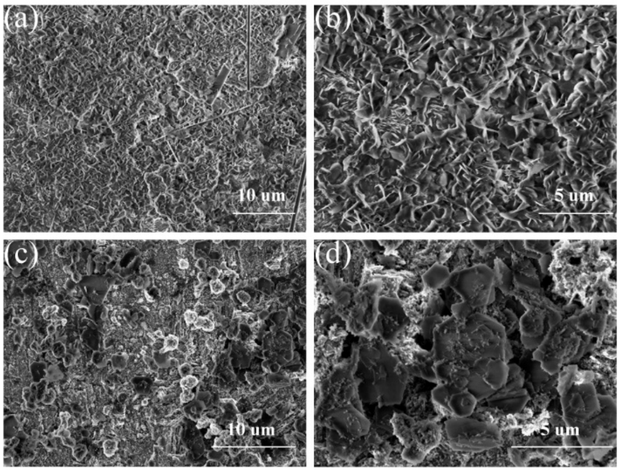

Figure S6. Surface morphology of bare Zn (a-b) and ZOCC@Zn (c-d) electrodes after 15 cycles at a current density of 0.5 mA cm^{-2} and a capacity of 0.25 mA h cm^{-2}, respectively

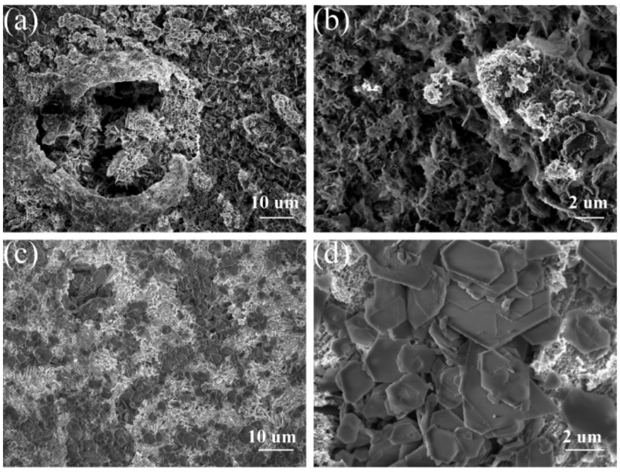

Figure S7. Surface morphology of bare Zn (a-b) and ZOCC@Zn (c-d) electrodes after 15 cycles at a current density of 1 mA cm^{-2} and a capacity of 1 mA h cm^{-2}, respectively

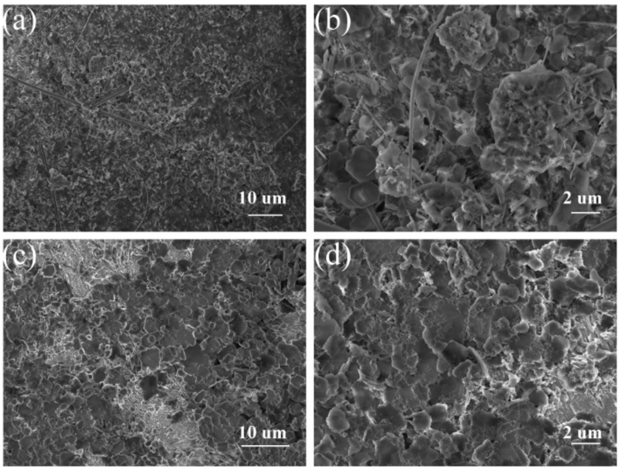

Figure S8. Surface morphology of bare Zn (a-b) and ZOCC@Zn (c-d) electrodes after 15 cycles at a

current density of 5 mA cm-2 and a capacity of 1 mA h cm-2, respectively

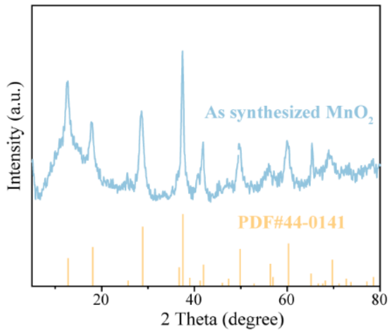

Figure S9. The XRD pattern of α-MnO_2

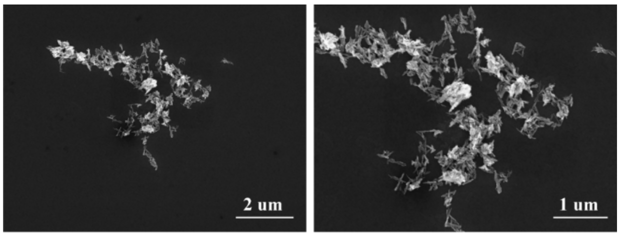

Figure S10. SEM pattern of α-MnO_2 at different magnifications

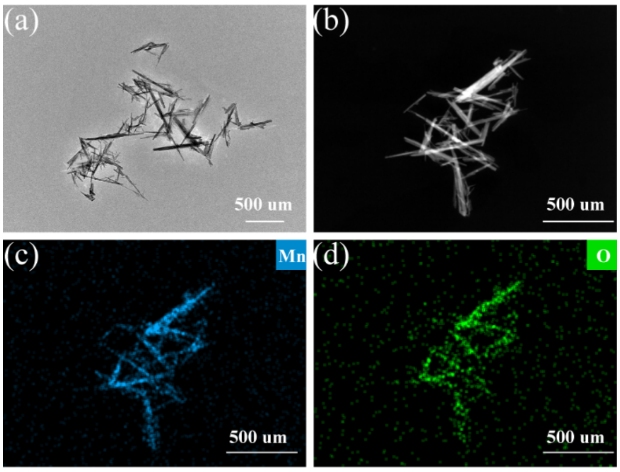

Figure S11. (a) TEM image of α-MnO_2; (b) HAADF-STEM image of α-MnO_2; Corresponding elemental mapping of (c) Mn and (d) O

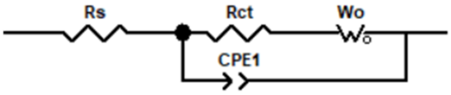

Figure S12. The equivalent circuit used for fitting the experimental EIS data
